# Characterization of *Bacillus velezensis* LH023 Isolated From Grass Carp Intestines and Its Beneficial Effects on Growth, Immunity, and Gut Microbiota in Aquaculture

**DOI:** 10.1155/anu/5594265

**Published:** 2025-06-29

**Authors:** Lihan Liu, Jiaming Huang, Yiling Huang, Zhendong Qin, Fubin Zhang

**Affiliations:** ^1^College of Environmental Science and Engineering, China West Normal University, Nanchong 637009, China; ^2^Guangdong Evergreen Feed Industrial Co., Ltd., Zhanjiang 524002, China; ^3^Zhongkai University of Agriculture and Engineering, Guangzhou 510225, Guangdong, China

**Keywords:** *Bacillus velezensis*, *C. idella*, immunoregulation, intestinal flora, intestinal health

## Abstract

In this study, *Bacillus velezensis* was isolated from the intestines of grass carp and identified as LH023 via 16S rRNA sequencing and morphological analysis. To evaluate its potential as a feed additive in aquaculture, LH023 was supplemented at two concentrations (10^7^, 7 × 10^7^ and 10^8^, 7 × 10^8^ CFU/g) in a 6-week feeding trial, alongside a control group. Enzyme activity assays demonstrated that LH023 supplementation enhanced growth performance and increased intestinal enzyme levels, including α-amylase, lipase, protease (Pro), and trypsin. Real-time quantitative PCR (qRT-PCR) analysis revealed significant downregulation of pro-inflammatory genes (*IFN-γ2, c-Rel, NF-κB, p52, IKKβ*, and *IKKγ*) in intestinal tissue, while anti-inflammatory cytokine genes (*S6K1, IL-4*, and *IL-10*) were upregulated. Additionally, antioxidant-related genes (*CAT, SOD, GSH*, and *Keap1a*) and intestinal barrier markers (*ZO-2, ZO-3*, and *claudin-12*) were significantly upregulated in the LH023 treatment groups. 16S rRNA sequencing revealed that the 10^7^ and 10^8^ groups exhibited a higher abundance of beneficial microbiota, including Clostridia, Gammaproteobacteria, Bacteroidia, Deltaproteobacteria, and Bacilli. KEGG pathway analysis identified enrichment in metabolic pathways related to carbohydrate metabolism, cofactor and vitamin metabolism, amino acid metabolism, and lipid metabolism. α-Diversity analysis showed no significant differences in microbial diversity (*p*  > 0.05), while β-diversity analysis indicated that the microbial communities in the 10^7^ and 10^8^ groups were distinct from the control, with low inter-group similarity. Histological examination (hematoxylin–eosin (HE) and periodic acid–Schiff (PAS) staining) of intestinal, liver, and kidney tissues demonstrated that LH023 supplementation effectively mitigated the tissue damage caused by *Aeromonas hydrophila* infection and significantly improved grass carp survival. These results suggest that *B. velezensis* LH023 holds promise as a beneficial probiotic in aquaculture.

## 1. Introduction

Grass carp (*Ctenopharyngodon idella*) belongs to the bony fish family Cypriniformes, Cyprinidae, and is one of the four main fish in China, along with black carp, silver carp, and bighead carp [1, 2]. In recent years, improvements in the diet structure and the continuous expansion of the demand for aquatic products have resulted in the vigorous development of China's aquaculture industry. Grass carp is an important breed in freshwater aquaculture due to advantages such as mature breeding technology, low breeding costs, high survival rates, abundant yields, and straightforward management. According to the 2023 China Fishery Statistical Yearbook, the aquaculture output of grass carp in 2023 in the country exceeded 5.9 million tons, ranking first in China's freshwater fish aquaculture output. In terms of culture area, the total amount of grass carp culture varies among different provinces [3]. However, the profitability of grass carp breeding is challenged by the rising costs of feed, stringent animal protection requirements, and the labor involved in artificial breeding processes.

With the development of highly intensive farming models, aquaculture animals are threatened by serious diseases caused by viruses, bacteria, fungi, parasites, algae, and other undiagnosed pathogens [4]. A breakout of bacterial infectious diseases can easily lead to the high mortality of aquaculture animals, resulting in serious economic losses [5]. Common bacterial diseases in aquaculture include rotten gill disease, erythroderma, enteritis, and bacterial septicemia of freshwater fish. These diseases can lead to the slow growth and increased mortality of aquatic animals. Antibiotics are widely used in aquaculture to solve the problem of diseases and improve production efficiency. However, antibiotic residues and bacterial drug resistance have become global problems due to the widespread abuse of antibiotics. To address the issue of antibiotic pollution, there is a demand to determine alternative technologies and develop antibiotic substitutes [6].

Among several substitutes for antibiotics, the use of probiotics is an important approach to achieving green aquaculture and is considered a safe and unconventional alternative [7]. Probiotics are living microorganisms that exert positive effects on the health of aquatic animals, for example, related to immunity, digestion, and development, when consumed in sufficient quantities [8]. Guo et al.'s research on *Bacillus subtilis* supplementation in grass carp feed demonstrated its potential anti-inflammatory effects through the detection of immune index measurements such as IL-10 and TGF-β [9]. Sadat Hoseini Madani et al. [10] revealed that the probiotic diet enhanced growth performance parameters, serum biochemical parameters, blood cell counts, and improved the immune parameters of white-legged shrimp.

*Bacillus velezensis* belongs to a new species of *Bacillus*. It is a gram-positive aerobic bacterium with a rod-shaped body and is widely distributed in natural water, soil, air, plant roots and surfaces, and animal intestines. The bacterium was first isolated by Ruiz-García et al. [11] from the mouth of the Velez River in the Torre del Mar region of Malaga, Spain. At present, research on the application of spore probiotics as feed additives in aquaculture mainly employs *B. subtilis* as the principal feed additive. The majority of studies on *B. velezensis* focus on water quality regulation, drug development, and food fermentation, or use the bacterium as a microbial agent for plant research. However, reports on the use of *B. velezensis* as a feed additive for aquatic animals are limited.

In this study, a strain of *B. velezensis* was isolated from the intestinal tract of healthy grass carp and named LH023. To evaluate the potential application of LH023 in aquaculture, the isolated *B. velezensis* was administered as a feed additive to grass carp for 6 weeks. Growth performance parameters, immune indices, intestinal flora, and histopathological changes in the grass carp were assessed. The results provide a theoretical basis for the healthy cultivation of grass carp.

## 2. Materials and Methods

### 2.1. Experimental Fish and Sampling

The experimental grass carp (49–55 g) used in this study were purchased from the Guangzhou grass carp breeding base (in Guangdong province), transported back to the laboratory, and temporarily raised in a circulating aerated water tank in the fish house laboratory. The fish were fed in the morning and evening every day to ensure sufficient oxygen. The experiment was performed following a 2-week adaption period for the experimental grass carp. The fish were randomly divided into control, 10^7^ (7 × 10^7^ CFU/g), and 10^8^ (7 × 10^8^ CFU/g) groups, with 150 fish in each group. All grass carps were fed daily at 9:00 and 16:00 and sampled after 6 weeks of feeding.

### 2.2. Isolation and Identification of *Bacillus*

Under aseptic conditions, the anterior, middle, and posterior sections of the intestine of healthy grass carp were sampled and divided into centrifuge tubes, and thoroughly mixed with sterile normal saline. After a water bath at 60°C for 20 min, the mixture was centrifugated at 3000 rpm for 5 min. Following this, 100 μL of supernatant was added to an LB plate and cultured at 28°C for 1–2 days. A single colony was selected from the plate with an inoculation ring and purified three times.

### 2.3. Molecular Identification

The single colony purified by the third scribing was selected and cultured in 1 mL LB liquid medium at 28°C and 200 rpm in a shaking table for 4 h. PCR amplification of 16S rDNA and *gyrB* gene sequencing was then performed.  Table 1 reports the sequences of common primers. The PCR products were sequenced by Guangzhou Tianyi Huiyuan Gene Technology Co., Ltd (Guangzhou, China). Sequence analysis using the GenBank database showed that the strain LH023 was 99.65% similar to *B. velezensis*. Considering that *B. velezensis* and *B. subtilis* are closely related and have similar phenotypes, 12 strains with high sequence homology were selected from the database for further study. A phylogenetic tree was constructed by the neighbor joining method in MEGA 11.0 (Pennsylvania State University), and the reliability of the tree was evaluated using bootstrapping with 1000 repetitions.

### 2.4. Gram Staining

The colonies or bacterial sheets picked by the toothpick were evenly mixed and spread on a slide with a small amount of sterile water. The crystal violet dyeing solution was added and incubated for 2 min, followed by the staining of gram iodine solution for 2 min. After washing the slide with water, a decolorizing solution was added and shaken for 30 s to terminate the reaction. Finally, the slide was observed under a microscope after incubation with drop Gaza yellow dye for 60 s.

### 2.5. Morphological Identification

After the LH023 was cultured on an LB solid plate overnight at 28°C, a single colony was placed in 1 mL LB medium and cultured at 28°C for 4 h, then transferred to 5 mL LB medium for another 6 h. Following the centrifugation of the bacterial solution at 2000 rpm for 5 min, the pellet was fixed with 1 mL of 2.5% glutaraldehyde fixing solution and sent to Wuhan Sewili Biotechnology Co., Ltd (Wuhan, China) for electron microscope scanning observations.

### 2.6. Physiological and Biochemical Identification

The LH023 was added to a microbial reaction tube (purchased from Hangzhou Binhe Microbial Company, Hangzhou, China) and placed in a 28°C incubator for 12 h. Physiological and biochemical identification was performed according to Berger's Manual of Bacterial Identification (8th edition) and the Manual of Common Bacterial System Identification [12].

### 2.7. Survival Curve Identification

The LH023 was cultured in 1 mL of LB medium and incubated in a shaking table at 28°C and 200 rpm overnight at constant temperature. When the OD_600_ value of LH023 was 0.4–0.6, the bacteria was transferred into a conical bottle containing 100 mL LB medium. The OD value of LH023 was measured every 2 h for a total of 20 h after constant temperature cultivation in a shaking table at 200 rpm and 28°C.

### 2.8. Bacteriostatic Identification

*Aeromonas hydrophila* and *Staphylococcus aureus* were selected to detect the antagonistic activity of LH023 by the Oxford cup method. Briefly, 1 × 10^5^ CFU of *S. aureus* and *A. hydrophila* were cultured on an LB plate, and 200 μL of LH023 was added to an Oxford cup. The PBS and gentamicin were used as the negative and positive control, respectively, and the plates were incubated at 28°C in an incubator overnight.

### 2.9. Drug Sensitivity Test Identification

The drug sensitivity of LH023 was determined by the disk diffusion method (K-B) [13]. Briefly, 200 μL of LH023 was absorbed and coated on an LB nutrient agar plate. The drug-sensitive paper (obtained from Hangzhou Tianhe Microbial Reagents Co., Ltd, Hangzhou, China) was attached to the plate and cultured at 28°C for 24 h. Referring to the product description of the drug-sensitive tablets, the drug resistance of the bacteria was determined by the standards in the literature [14] and the product description of Hangzhou Tianhe Microbial Reagents Co., Ltd. In particular, the following thresholds were employed according to the inhibition zone diameter: inhibition zone diameter ≥17 (mm) denotes high sensitivity; 14 ≤ inhibition zone diameter <17 (mm) denotes medium-to-high sensitivity; and inhibition zone diameter <14 (mm) denotes drug resistance.

### 2.10. Enzyme Activity Detection Index

Malondialdehyde (MDA), superoxide dismutase (SOD), protease (Pro), glutathione peroxidase (GSH-Px), catalase (CAT), alkaline phosphatase (AKP), cellulase (CE), trypsin, lipase, and α-amylase assay kits were used to detect antioxidant enzymes and damage-related enzyme activity according to the manufacturer's guidelines.

### 2.11. Real-Time Fluorescent Quantitative PCR

Real-time quantitative PCR (qRT-PCR) analysis was conducted to investigate the effect of probiotics supplementation on the related immune indexes of grass carp. The total volume of qRT-PCR amplification was 20 μL, including 4 μL of cDNA, 5 μL of water, 0.5 μL of each gene-specific primer (10 μM), and 10 μL of AceQ qPCR SYBR Green Master Mix (Vazyme, Nanjing, China). The reaction was performed using a qTOWER3 fluorescence quantitative PCR instrument (Analytik Jena AG, Germany), with the following reaction procedure: pre-denatured at 95°C for 30 s, 95°C for 10 s, and 60°C for 30 s. The primer specificity was verified by a melting curve, and the relative expression ratio of the target genes versus β-actin was calculated using the 2^−*ΔΔ*Ct^ method.

### 2.12. Intestinal Flora Sequencing

The intestines of grass carp were collected and sent to Shenzhen BGI Co., Ltd. (China) for 16S sequencing. The 16S sequencing data was then used to perform taxa clustering, α/β-diversity analysis, and KEGG analysis and to determine OTU abundance, heat maps, and species column graphs. The Simpson, ACE, Chao, and Shannon indexes were used to analyze the gut microbiome *α*-diversity. The unweighted-UniFrac and binary Jaccard algorithms were employed for β-analysis diversity. QIIME was used to perform principal coordinate analysis of the primary coordinates and to create the thermal cluster graphs.

### 2.13. Grass Carp Attack Experiment

The experimental grass carp were divided into two experimental groups and a control group. The experimental groups were injected with *A. hydrophila* solution at a concentration gradient of 5 × 10^4^ CFU/mL, while the control group was injected with the same volume of saline. The survival rate was counted for 1 week, and the infection symptoms and number of deaths of the test fish were recorded for seven consecutive days.

### 2.14. Hematoxylin–Eosin (HE) and Periodic Acid–Schiff (PAS) Staining of Intestinal Tissue

To explore the effect of probiotics feeding on the intestinal structure of grass carp, the tissues were collected for HEPAS staining. Briefly, after treatment with xylene anhydrous ethanol Ⅰ for 5 min, anhydrous ethanol Ⅱ for 5 min, and 75% alcohol for 5 min, the intestinal tissue sections were stained with hematoxylin dye solution for 5 min. After differentiation and blue return steps, the sections were treated with 85% and 95% gradient alcohol and dehydrated for 5 min, respectively. The eosin dye solution was used to stain the slices for 5 min, following treatment with anhydrous ethanol and xylene gradient for 5 min. For PAS staining, the slices were stained in PAS staining solution B for 15 min and subsequently immersed in PAS staining solution A for 30 min. This was followed by immersion in PAS staining solution C for 30 s. Finally, the samples were observed with a microscope and images were collected.

### 2.15. Data Analysis

All data were expressed as the mean ± SD (*n* = 6). Significant differences were analyzed by one-way ANOVA in SPSS. 24.0 (IBM, SPSS Inc.). Differences were considered significant and extremely significant for *p* < 0.05 and *p* < 0.01, respectively. The results were plotted in GraphPad Prism 9 (GraphPad Software, Inc.).

## 3. Results

### 3.1. Screening of the Potential Probiotics in the Intestinal Tissue of Grass Carp

**Figure 1:**
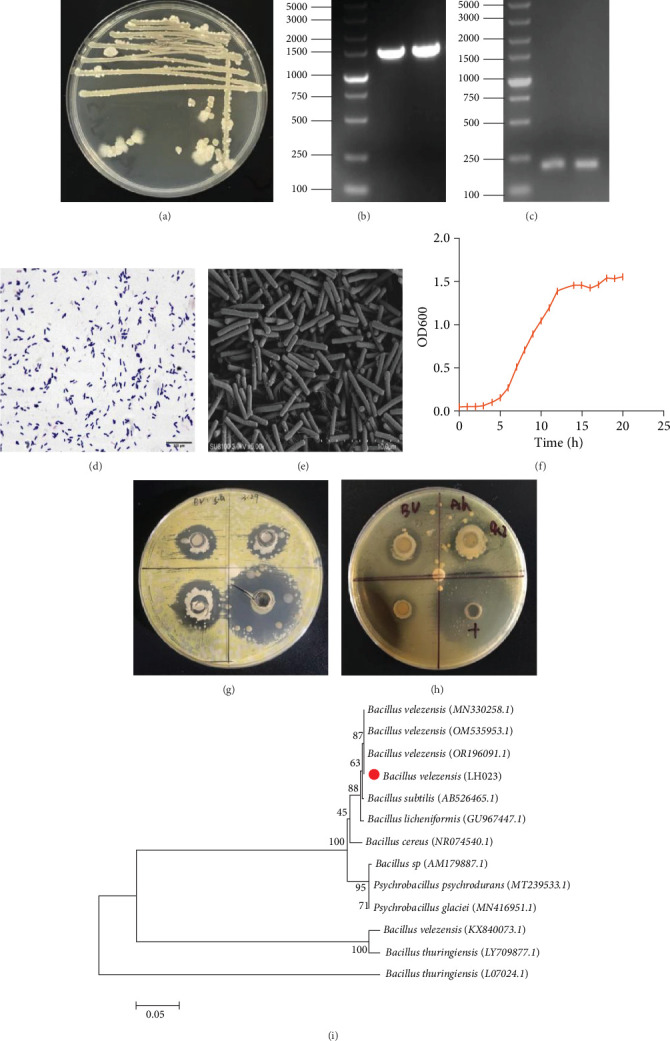
Screening of potential probiotics in grass carp gut. (A) Colony morphology of strain LH023. (B) Electrophoresis of primer 16S-27-F PCR amplification products. (C) Electrophoretic image of PCR amplification product with *gyrB* primer. (D) Gram staining result of strain LH023. (E) SEM results of strain LH023 (Seville, Wuhan). (F) Growth curve of strain LH023. (G) Effect of strain LH023 inhibiting *Staphylococcus aureus*. (H) Effect of strain LH023 inhibiting *Aeromonas hydrophila*. (I) Phylogenetic analysis of *Bacillus velezensisensis* LH023.

We investigated the colony morphology of strain LH023 isolated from the intestinal tract of healthy grass carp cultured on LB medium. The colony was observed to be milky white, opaque, and sticky, with rough and wet surfaces, and irregular and raised edges (Figure 1A). The PCR amplification of 16S rDNA (Figure 1B) and *gyrB* gene sequence (Figure 1C) determined sequence sizes of 1500 and 150 bp, respectively, with a GenBank entry number of OR272311. BLAST analysis showed that the 16S rRNA sequence of the strain had a 99.65% homology with *B. velezensis*, indicating a high degree of similarity between the two (Figure 1I). Gram staining (Figure 1D) and electron microscopy (Figure 1E) showed that the strain was Gram-positive, and the bacteria grew straight rods (single or multiple) that could form spores. Combined with the external morphological observation of the strain, the strain was identified as *B. velezensis*, and named LH023. The strain has been stored in the China Microbiological Culture Preservation Center with the storage number CGMCC28417.  Table 2 lists its physiological and biochemical test results. LH023 tested negative for indole, methyl red, mannitol, and Simon's citrate, but tested positive for aesculin, gelatin, and other tests. Furthermore, LH023 was able to ferment sucrose, cellobiose, and inulin broth, but could not decompose xylose, arabinose, raffinose, lactose, and other sugars. The growth curve (Figure 1F) indicates that LH023 had a growth retardation period of 0–5 h, where the bacteria were in a stage of environmental adaptation, exhibiting a slow growth rate. A logarithmic growth stage was observed at 5–12 h, whereby the bacteria proliferated rapidly. From 12–20 h, the bacterial propagation rate was moderate. *Bacillus velezensis* had the strongest vitality, the highest growth rate, and the most vigorous metabolic activity from 5 to 12 h. The results of the drug sensitivity test showed (Table 3) that *B. velezensis* was sensitive to 18 antibiotics, including cefalexin, ceftriaxone, cefradine, and enoxacin (S), and demonstrated tolerance to lincomycin (R). The results of the bacteriostatic experiment revealed that the diameter range of the three bacteriostatic circles of *S. aureus* was 11–17 mm (Figure 1G), and that of *A. hydrophila* was 15–20 mm (Figure 1H).

### 3.2. Diet of LH023 Improved the Growth Performance and Immunity of Grass Carp

**Figure 2:**
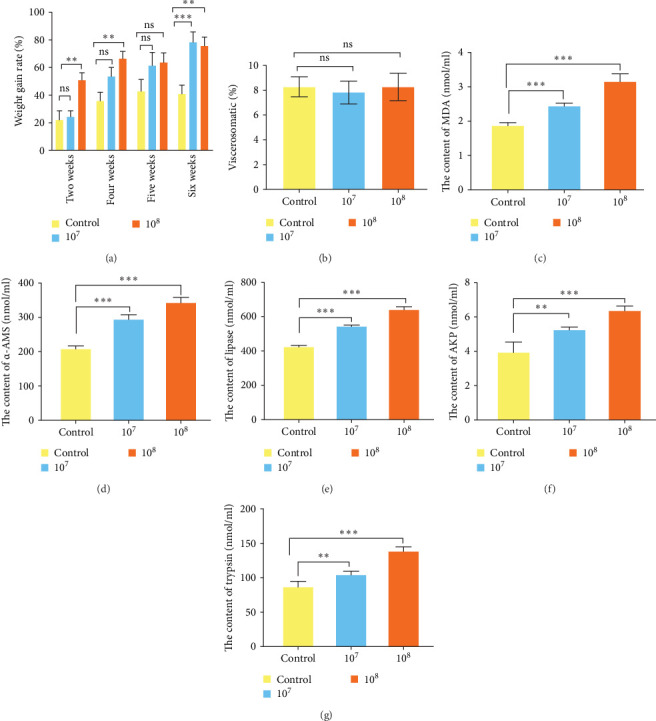
Effects of weight gain rate, viscera body ratio, and strain LH023 on intestinal enzyme activity of grass carp. (A) After feeding strain LH023, grass carp gained weight in Week 2, Week 4, Week 5, and Week 6. (B) Results of splanchnosomatic ratio of grass carp at week 6 after feeding strain LH023. (C) MDA: malondialdehyde. (D) α-AMS: α-amylase. (E) Lipase: lipase. (F) AKP: alkaline phosphatase. (G) trypsin: trypsin. The data were the average of three independent experiments, expressed by mean ± SEM (*⁣*^*∗*^*p* < 0.05, *⁣*^*∗∗*^*p* < 0.01, *⁣*^*∗∗∗*^*p* < 0.001).

To investigate the effects of LH023 on the growth performance of grass carp, we performed weight gain analysis.  Figure 2A showed that the body weight of the 10^8^ group increased significantly in the second, fourth, and sixth weeks (*p* < 0.01), and that of the 10^7^ group increased significantly in the sixth week (*p* < 0.001).  Figure 2B indicates that there were no significant differences in VSI between groups 10^7^ and 10^8^ in the fifth week (*p* > 0.05). After feeding grass carp with LH023 for 6 weeks, the contents of MDA (Figure 2C), α-AMS (Figure 2D), and lipase enzyme activities (Figure 2E) in the intestinal tissues of groups 10^7^ and 10^8^ increased significantly compared to those of the control group (*p* < 0.001). In addition, the contents of AKP (Figure 2F), and trypsin (Figure 2G) were increased significantly in group 10^7^ (*p* < 0.01) and increased extremely significantly in group 10^8^ (*p* < 0.001).

### 3.3. Diet of LH023 Regulated the Expression of Inflammatory Factors in Intestinal Tissues

**Figure 3:**
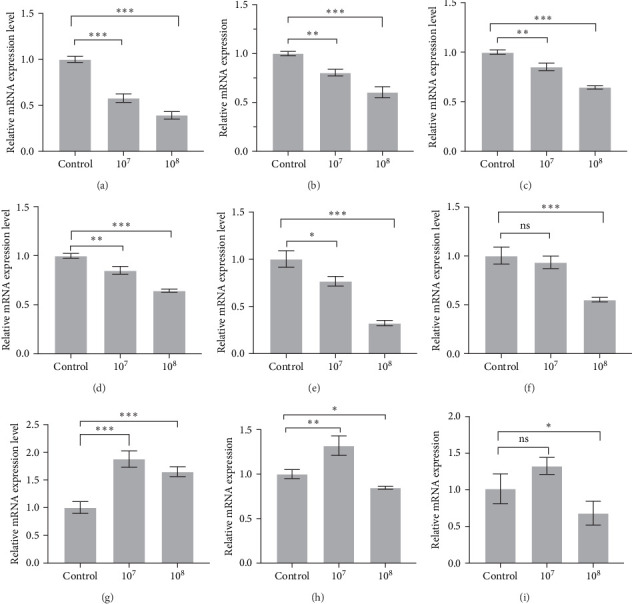
Strain LH023 participated in the regulation of intestinal inflammatory factors in grass carp. (A) *c-Rel* gene. (B) *TLR-4* gene. (C)*NF-кBp52* gene. (D) The *IKKβ* gene. (E) *IFN-γ2* gene. (F) The *IKKγ* gene. (G) *S6K1* gene. (H) *IL-4* gene. (I) *IL-10* gene. The data were the average of three independent experiments, expressed by mean ± SEM (*⁣*^*∗*^*p* < 0.05, *⁣*^*∗∗*^*p* < 0.01, *⁣*^*∗∗∗*^*p* < 0.001).

qRT-PCR was performed to detect the effect of supplementing LH023 on the inflammatory response in intestinal tissues. As shown in Figure 3 that the expression levels of genes *c-Rel*, *TLR4*, *NF-кBp52*, *IKKβ*, *IFN-γ2*, and *IKKγ* decreased significantly in group 10^8^ after the sixth week of LH023 feeding (*P* < 0.001). In group 10^7^, there was a significant decrease in the expression levels of *c-Rel* (*P* < 0.001), *TLR4*, *NF-кBp52*, and *IKKβ* (*p* < 0.01), while no significant differences were observed in the *IKKγ* expression level (*p* > 0.05). The expression level of anti-inflammatory factor *S6K1* increased significantly in groups 10^7^ and 10^8^ (*p* < 0.001), while the *IL-4* and *IL-10* expression levels also increased significantly (*p* < 0.05) in group 10^8^. The expression of *IL-4* in group 10^7^ increased significantly (*p* < 0.01), while no significant differences were observed for the *IL-10* expression level (*p* > 0.05).

### 3.4. Diets of LH023 Modulated the Inflammatory Response in the Grass Carp

**Figure 4:**
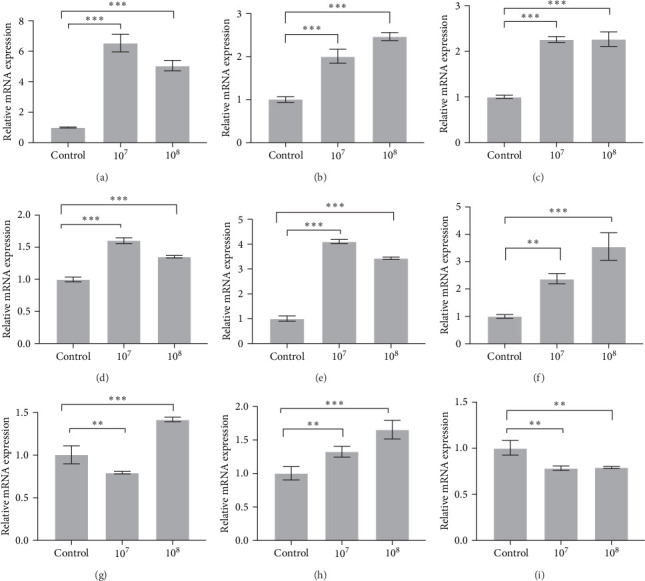
Strain LH023 regulates proinflammatory gene expression in head kidney of grass carp. (A)*TNF-α* gene. (B) *IL-15* gene. (C) *IL-8* gene. (D) *NF-кBp52* gene. (E) *IL-17D* gene. (F) *IL-6* gene. (G) *TLR-4* gene. (H) *c-Rel* gene. (I) *IKKγ* gene. The data were the average of three independent experiments, expressed by mean ± SEM (*⁣*^*∗*^*p* < 0.05, *⁣*^*∗∗*^*p* < 0.01, *⁣*^*∗∗∗*^*p* < 0.001).

As shown in Figure 4, the expression levels of *TNF-α*, *IL-15*, *IL-8*, *NF-кBp52*, and *IL-17D* in groups 10^7^ and 10^8^ increased significantly (*p* < 0.001). The expression levels of *TLR-4* exhibited a downward trend in group 10^7^ (*p* < 0.01) and an upward trend in group 10^8^ (*p* < 0.001). The expression levels of *IL-6* and *c-Rel* increased significantly in groups 10^7^ (*p* < 0.01) and 10^8^ (*p* < 0.001), however, those of *IKKγ* decreased in both groups (*p* < 0.01).

### 3.5. Diet of LH023 Increased the Expression of Antioxidant Factors and the Mucosal Barriers of Intestinal Tissues

**Figure 5:**
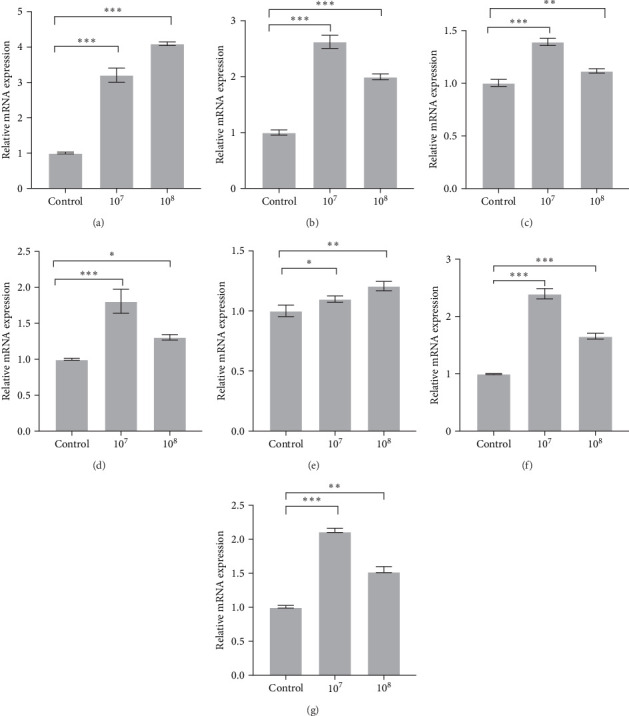
Strain LH023 regulates intestinal antioxidant and intestinal mucosal barrier related genes in grass carp. (A) *CAT* genes. (B) *SOD* gene. (C) *GSH* gene. (D) *Keap1a* gene. (E) *claudin-12* gene. (F) *ZO-2* gene. (G) *ZO-3* gene. The data were the average of three independent experiments, expressed by mean ± SEM (*⁣*^*∗*^*p* < 0.05, *⁣*^*∗∗*^*p* < 0.01, *⁣*^*∗∗∗*^*p* < 0.001).

To evaluate the regulation function of supplementing LH023 on antioxidant factors and the mucosal barrier, we detected the expression of related genes. After 6 weeks of supplementing LH023, the transcription levels of *CAT* (Figure 5A), *SOD* (Figure 5B), *GSH* (Figure 5C), and *Keap1ɑ* (Figure 5D) in the intestinal gene significantly increased in groups 10^7^ and 10^8^ compared with the control (*p* < 0.001). The expressions of mucosal barrier-related genes (e.g., *claudin-12*, *ZO-2*, and *ZO-3*) were also upregulated in groups 10^7^ and 10^8^ (Figure 5).

**Figure 6:**
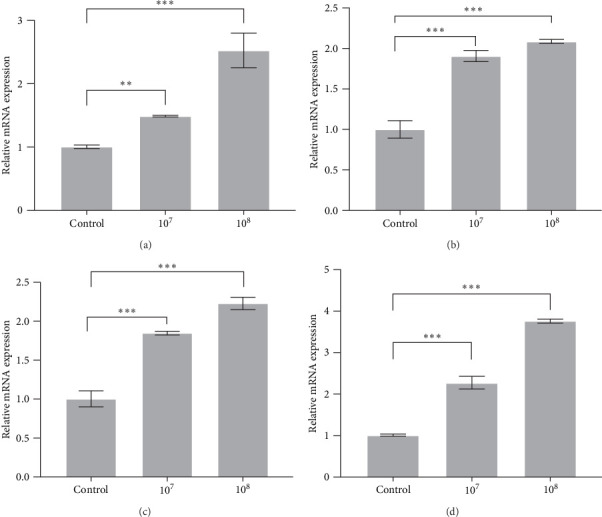
Strain LH023 regulates genes related to antioxidant and anti-inflammatory factors in head kidney of grass carp. (A) *GSH* gene. (B) *CAT* gene. (C) *IL-4* gene. (D) *IL-10* gene. The data were the average of three independent experiments, expressed by mean ± SEM (*⁣*^*∗*^*p* < 0.05, *⁣*^*∗∗*^*p* < 0.01, *⁣*^*∗∗∗*^*p* < 0.001).

The expressions of antioxidant factors and anti-inflammatory factors in head kidney-related genes were also detected by qRT-PCR. The detection results revealed that supplementing LH023 upregulated the expression of *GSH* and *CAT* genes in the head kidney in groups 10^7^ and 10^8^ (Figure 6A, B). Similarly, the expression levels of *IL-4* and *IL-10* in the head kidney significantly increased in groups 10^7^ and 10^8^ (Figure 6C, D).

### 3.6. Diet of LH023 Changed the Species Composition and Microbial Community

**Figure 7:**
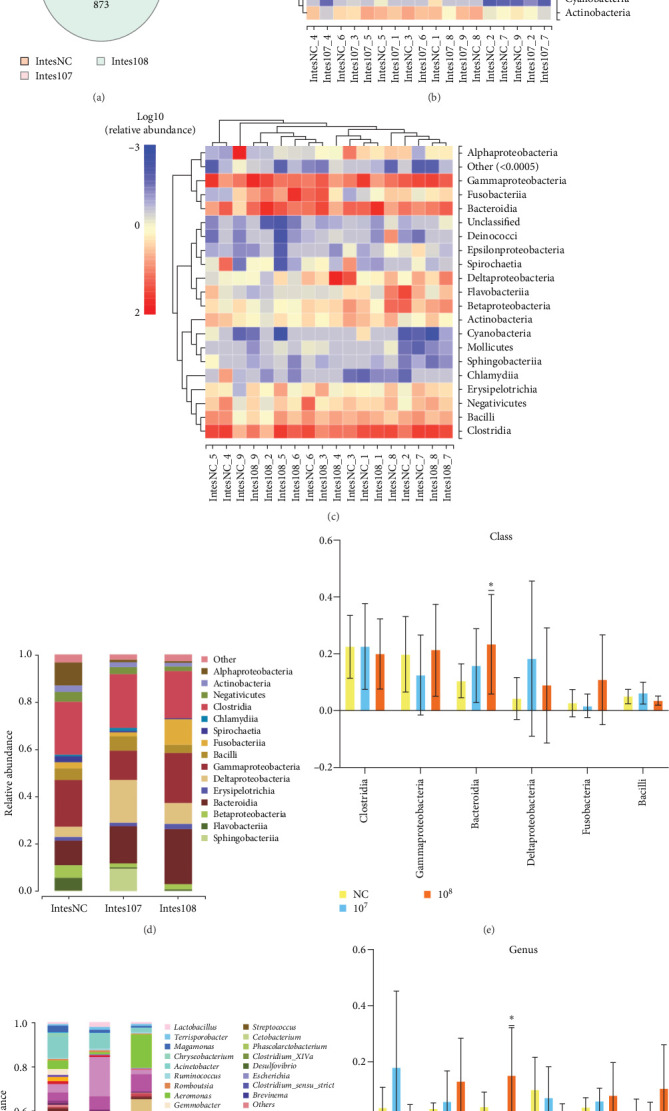
Analysis of intestinal microbes in different treatment groups by Wayne diagram, heat map, and species column diagram. (A) Venn Diagram. (B) Heat maps of 10^7^ groups of intestinal microorganisms at class level. (C) Heat maps of 10^8^ groups of gut microbes at class level. (D, E) Species histogram of gut microbes in different treatment groups at class level. (F, G) Species histogram of gut microbes in different treatment groups at genus level.

To further explore the effects of LH023 on the intestinal flora of grass carp, 16S sequencing was employed to evaluate the intestinal tract. The Venn diagram in Figure 7A shows that a total of 4648 OTUs were produced in the three groups, of which 810 were shared. A heat map was produced using the first 21 samples of bacterial abundance similarity. A comparison of major bacterial classes in the gut of the two groups showed that the core flora were similar on the class level, including Clostridia, Bacilli, Negativicutes, Gammaproteobacteria, Bacteroidia, Deltaproteobacteria, and 21 other species of bacteria (Figure 7B, C). We determined the species histogram at the class and genus levels based on the top 15 and 31 species of relative abundance in the bacterial community, while the remaining classes and genera with low abundance were classified as “others” (Figure 7). The first six species of each classification were included in the histogram. The results show that at the class level, Clostridia, Gammaproteobacteria, Bacteroidia, Deltaproteobacteria, Fusobacteriia, and Bacilli are abundant (Figure 7E). At the genus level, *Lawsonia*, *Bacteroides*, *Aeromonas*, *Acinetobacter*, *Prevotella*, and *Cetobacterium* are abundant in six genera (Figure 7G).

### 3.7. Effects of LH023 on the Key Species of the Intestinal Microorganisms of Grass Carp

**Figure 8:**
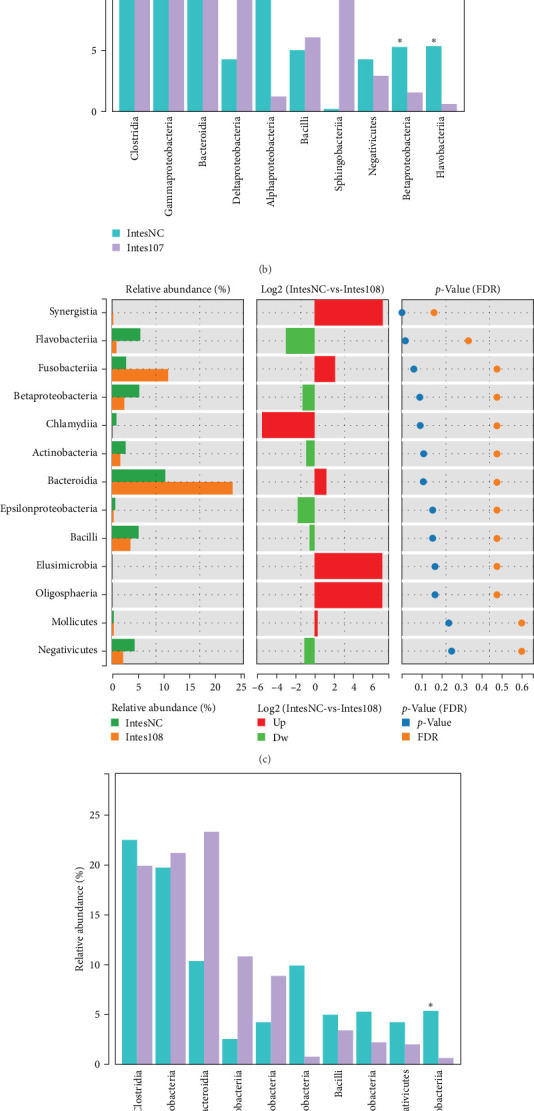
Comparison of intestinal microbial key species in different treatment groups and analysis of KEGG results. (A) Intestinal microbial species difference analysis diagram of group 10^7^. (B) Comparison map of key species of intestinal microbes in group 10^7^. (C) Species difference analysis diagram of 10^8^ groups of intestinal microbes. (D) Comparative map of key species of intestinal microbes in 10^8^ groups. (E) KEGG results of intestinal microbes in different treatment groups.

To further explore the effects of LH023 on the key species of grass carp gut microbes, statistical indicators and the false discovery rate (FDR) were used to evaluate the significant differences in crop species. In addition, the top 10 species in abundance were selected to indicate the average relative abundance of each group and the significant differences test for key species comparison. The results showed that at the class level, the abundance of Betaproteobacteria, Flavobacteriia, and other bacteria in experimental group 10^7^ exhibited a decreasing trend (*p* < 0.05) (Figure 8A, B). In experimental group 10^8^, only Flavobacteriia showed a decreasing trend (Figure 8C, D). Enrichment analysis of the KEGG pathway was then performed on the bacterial strain function of the control, 10^7^, and 10^8^ groups. The results showed that the top seven enriched metabolic pathways were carbohydrate metabolism, metabolism of cofactors and vitamins, amino acid metabolism, metabolism of cofactors and vitamins, metabolism of terpenoids and polyketides, metabolism of other amino acids, and lipid metabolism (Figure 8E).

### 3.8. LH023 Modulated the Intestinal Microbial Community

**Figure 9:**
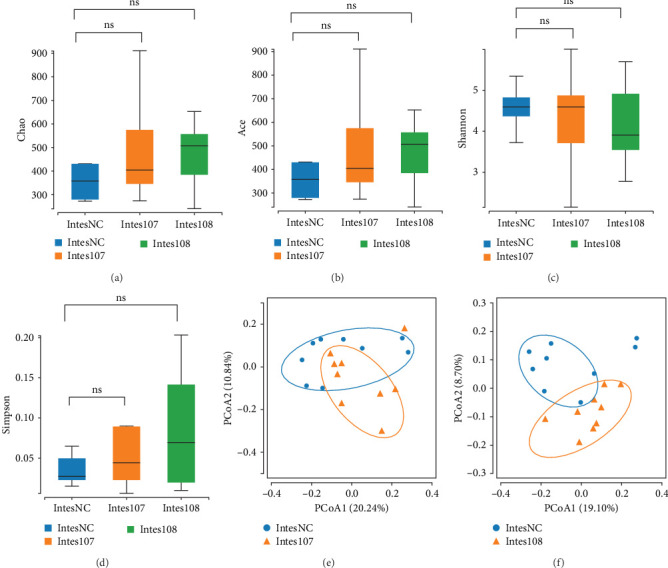
Results of αβ diversity analysis of intestinal microorganisms in different treatment groups. (A) Map of Chao index species richness results. (B) Plot of ACE index species richness results. (C) Shannon index species richness results. (D) Plot of Simpson index species richness results. (E) Results of PCOA analysis of 10^7^ groups. (F) Results of PCOA analysis of 10^8^ groups.

To further explore the effects of LH023 on the diversity of intestinal microbial communities of grass carp, α-diversity statistical analysis was performed. The Chao and ACE indices were employed to characterize the species richness, while the Shannon and Simpson indices were used to represent species diversity. After supplementing with LH023, there were no significant differences in the Chao, ACE, Shannon, and Simpson indexes in the intestinal tracts of grass carp in the two experimental groups compared with the control (Figure 9A–D). The β analysis results showed that groups 10^7^ and 10^8^ could be separated from the control group, indicating a high sample similarity within each group. The small number of shares between the groups indicated that the similarity between the groups was low (Figure 9E, F).

### 3.9. LH023 Maintained the Intestinal Health of Grass Carp

**Figure 10:**
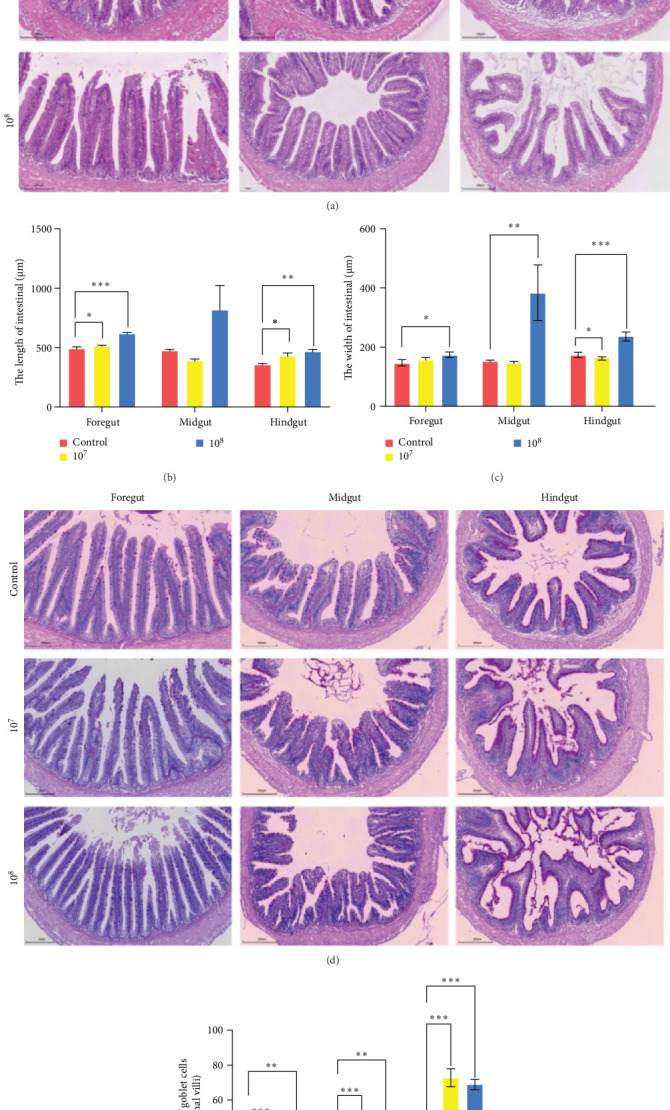
HE and PAS detection of the effects of different treatment groups on the intestinal tissue structure of grass carp. (A) Results of HE staining. (B) The length of intestinal villi. (C) The width of intestinal villi. (D) The PAS staining of intestinal tissue. (E) The number of goblet cells in the foregut, midgut, and hindgut. The data were the average of three independent experiments, expressed by mean ± SEM (*⁣*^*∗*^*p* < 0.05, *⁣*^*∗∗*^*p* < 0.01, *⁣*^*∗∗∗*^*p* < 0.001).

We further explored the immune protection effect of LH023 on the intestinal tract of grass carp. After *A. hydrophila* injection, the foregut, midgut, and hindgut were sampled for HE and PAS staining analysis. The HE staining results showed that the intestinal villi of the foregut and midgut had different degrees of rot and damage, while those in the 10^7^ and 10^8^ groups presented less damage (Figure 10A). The villus length and width in the foregut, midgut, and hindgut of the LH023 treatment group showed varying degrees of increase (Figure 10B, C). The PAS results showed that the number of goblet cells in the foregut, midgut, and hindgut of groups 10^7^ and 10^8^ successively increased (Figure 10D, E).

### 3.10. LH023 Improved the Resistance of Grass Carp Following *Aeromonas Hydrophila* Invasion

**Figure 11:**
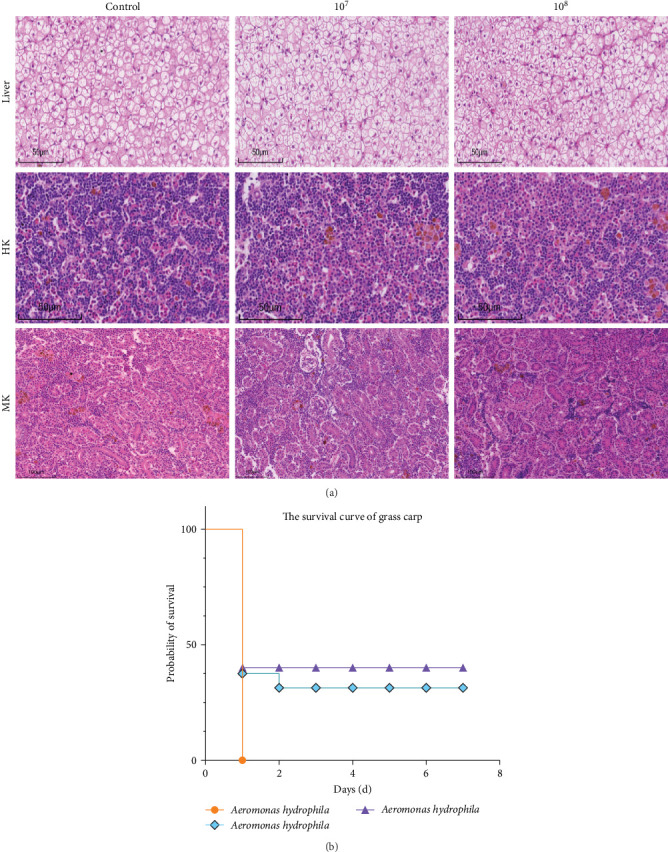
HE detection of the effects of different treatment groups on the tissue structure and survival rate of grass carp after challenge. (A) HE was used to detect the effects of challenge on liver, head kidney, and middle kidney of grass carp. (B) Survival curve of grass carp within 7 days after challenge.

To further understand the immunoprotective effect of LH023 on the organs of grass carp, an *A. hydrophila* challenge test was performed. The analysis through HE staining showed that after 6 weeks of LH023 feeding treatment combined with *A. hydrophila* infection, no significant structural differences were observed in the liver, mid-kidney, and head kidney (Figure 11A). Moreover, in the control group, all the grass carp died in the first day post-injection. The survival rate of group 10^7^ was 52% on day 1 and 48% on day 2, after which, no further deaths occurred. In group 10^8^, from the second day after infection of *A. hydrophila*, the mortality rate of the fish was maintained at 55% (Figure 11B).

## 4. Discussion

In this study, the *B. velezensis* isolated from the gut of healthy grass carp was identified by 16SrDNA, morphological observations, and physiological and biochemical identification, and named LH023. The colony was milky white, opaque, and sticky, with rough and wet surfaces and irregular and raised edges, and characterized as a gram-positive bacteria. The growth curve showed that at 28°C, the strain entered the logarithmic growth stage from 5 to 12 h, and essentially stabilized after 12 h. This agrees with the results of Liu et al. [15].

The bacteriostatic test showed that LH023 had an antagonistic ability against *S. aureus* and *A. hydrophila*, which is consistent with the results of Zhang and Wang [16] and Zhang et al. [17]. The drug sensitivity test indicated that LH023 was sensitive to 12 antibiotics such as cephalexin, norfloxacin, and erythromycin. LH023 may not carry antibiotic-resistance genes, and thus, *B. velezensis* presents adequate biosafety for practical applications. Heo et al. [18] evaluated the minimum inhibitory concentration of 123 strains of *B. velezensis* isolated mainly from fermented soybean foods in Korea. The authors revealed that all isolates were sensitive to chloramphenicol, clindamycin, erythromycin, gentamicin, kanamycin, tetracycline, and vancomycin, and it was suggested that *B. velezensis* was not at high risk for antibiotic resistance in food fermentation or human use [18]. This is consistent with the findings of our study.

In this study, the addition of LH023 isolated from the intestinal tract of grass carp as feed for 6 weeks was observed to significantly improve the growth performance indexes of grass carp. However, after the fifth week, the growth rate of grass carp in the 10^8^ group was lower than that in the 10^7^ group. This suggested that the probiotic concentration was too high and the grass carp had excess nutrition, which could slow down the grass carp growth. In a study by Hien, the growth rate, final body weight, and feed conversion rate of tilapia were significantly improved by the addition of *B. velezensis* isolated from the gut of tilapia [19]. This is similar to the results of this study.

The gut, an important digestive organ for fish, gathers a large number of microorganisms that are interdependent and interact with the host [20]. Through fermentation, *Bacillus* can enhance the activity of digestive enzymes in the body, promote the absorption of dietary nutrients by the intestinal tract of grass carp, and reduce the feed residue rate and the growth performance index of fish [21, 22]. Therefore, investigating the activity of digestive enzymes in the intestine is essential in detecting the digestion and absorption of fish feed by *B. belestre*. In this experiment, *α*-AMS, lipase, trypsin, Pro, AKP, and other enzyme activities of experimental groups 10^7^ and 10^8^ were higher than those of the control group. This indicates that LH023 could promote the activity of fish intestinal digestive enzymes, enhance the immune function of the body, and resist pathogenic microorganisms. Oxidative stress refers to the imbalance between oxidative and antioxidant functions in the body, which can cause damage by producing free radicals [23]. Probiotics can resist oxidative stress, which may be attributed to the ability of the probiotics to secrete antioxidant enzymes or promote the secretion of antioxidant enzymes in the body to improve the antioxidant capacity [24]. Researchers have reported MDA as a key substance generated by the attack of oxygen free radicals in the body on polyunsaturated fatty acids in the cell membrane. MDA can also reflect the degree of cell damage and the level of lipid peroxidation under free radical attacks. In serum, SOD and MDA typically have opposing enzyme activity content, and the two substances play complementary roles in the determination of blood indicators [25]. CAT, GSH-Px, and SOD play important roles in antioxidant stress-related processes in the body [26]. In this study, the enzyme activity contents of CAT, GSH-Px, and SOD in the intestinal tissues of grass carp fed with LH023 decreased, while the enzyme activity content of MDA products increased. This may be because LH023 damaged the antioxidant system in the intestinal tissues of grass carp.

The gut microbiota directly influences the balance of proinflammatory and anti-inflammatory responses in the gut [27]. Inflammation is a highly complex process and essentially a protective response of the body [2]. These biomolecules that promote the inflammatory response are known as proinflammatory factors [28]. Studies have shown that probiotics such as *Lactobacillus rhamnosus*, *Bifidobacterium*, and *Saccharomyces cerevisiae* Hansen can reduce the body's inflammatory response to improve immunity in animals [28, 29]. Probiotics or their products may reduce the production of inflammatory factors by inhibiting the NF-*к*B inflammatory signaling pathway [30]. In this study, feeding LH023 to grass carp inhibited the expression of proinflammatory factors such as *TLR4*, *NF-кBp52*, *IKK*β, and *IKK*γ in the gut of the NF-*к*B signaling pathway. A previous study added *B. velezensis* isolated from the intestines of zebrafish to feed together with nucleotides. The results revealed that the probiotic could reduce intestinal damage and inflammation caused by a high-fat diet by inhibiting proinflammatory factors (*NF-κB*, *TNF-α*, and *IL-1β*) [3]. Rhayat et al. [31] used the intestinal tract of broilers as an experimental model to analyze the NF-κB signaling pathway and evaluate the effect of *B. subtilis* on the intestinal tract. The authors showed that *B. subtilis* can reduce and prevent intestinal inflammatory response and strengthen the intestinal barrier [31]. This is consistent with the results of this study. Moreover, we found that strain LH023 activated the head renal proinflammatory factors *TNF-α*, *IL-15*, *IL-8*, *NF-кBp52*, *IL-17D*, *IL-6*, *TLR-4*, and *c-Rel* in experimental groups 10^7^ and 10^8^.

Reactive oxygen species (ROS) produced by metabolic processes in the body can help maintain normal physiological functions, and achieve a dynamic balance between the production and elimination of ROS through the antioxidant substances present in the body. Oxidative stress occurs when homeostasis is disrupted and has an impact on the survival, growth, development, and evolution of all living things [32]. Therefore, oxidative stress is also considered an important cause of various diseases. In this study, the qRT-PCR results showed that the supplementation of LH023 increased the gene expression levels of *CAT* and *GSH* in the gut and head kidney of grass carp, which may be caused by the depletion of the antioxidant system. Cheng revealed that adding 0.12%–0.18% *B. subtilis* to the diet could improve the immunity and antioxidant function of juvenile tilapia Gifu (*Oreochromis niloticu*) [33]. Maintaining a stable and healthy intestine is essential for a healthy body. The intestinal barrier can block the invasion of pathogenic bacteria, which helps to maintain the balance of the intestinal microbial community and plays a vital role in sustaining physical health [34]. *ZO-2* and *ZO-3* are intracellular tight junction proteins, and *claudin-12* is a transmembrane protein. The intestinal mucosa is tightly bound together by both transmembrane proteins and cytoplasmic proteins [35]. Probiotics can regulate the permeability of the intestinal mucosal barrier by regulating the expression of tight junction proteins [36]. In this study, the expression levels of the genes *ZO-2*, *ZO-3*, and *claudin-12* in the intestinal tract of grass carp significantly increased after the addition of LH023. This suggests that LH023 has the ability to reduce the permeability of intestinal mucosa, forming a protective barrier and improving the protective function of the intestinal tract.

Animal intestinal flora is diverse and complex, forming a symbiotic relationship with the host. Such parasitic and interdependent microorganisms create a special ecosystem that has a significant impact on the growth and development of their hosts [37]. In this study, after feeding grass carp with strain LH023, intestinal heat map analysis showed that Clostridia, Gammaproteobacteria, and Bacteroidia were present in the two experimental groups at the class level. Previous research reported that *Bacteroides* play a metabolic role in steroids, bile acids, and polysaccharides and promote polysaccharide absorption and protein synthesis. Moreover, Negativicutes are rich in beneficial bacteria, which promote intestinal energy absorption [38, 39]. Gammaproteobacteria belong to the Proteobacteria phylum, which in addition to containing a variety of pathogens, is also involved in the metabolism of a variety of bacteria [40]. Bacilli are generally beneficial bacteria that can inhibit harmful algae and aquatic pathogens in aquaculture [41]. Column chart analysis showed that in addition to the above classes, the abundance of clostridium and clostridium groups was also relatively concentrated. The dominant flora at the genus level is the genus *Aeroomonas*, belonging to Gammaproteobacteria, which is part of the intestinal microflora of healthy aquatic organisms. *Aeroomonas* may cause disease in fish and shrimp and even sudden death in extreme cases under certain environmental stresses. *Actinomyces*, a potential probiotic, is resistant to a variety of drug-resistant pathogenic microorganisms[42]. *Cetobacterium* can improve the immune index of fish and improve the health status of the body [43]. The class-level results of the comparison of key species showed that compared with the control group, the abundance of Flavobacteria in both experimental groups was significantly reduced. Our experiment also indicates that LH023 could inhibit the abundance of harmful pathogenic microorganisms in the intestinal tissue of grass carp. The enrichment of the KEGG signaling pathway showed that the top six signaling pathways were carbohydrate metabolism, cofactor and vitamin metabolism, terpenoid and polyketide metabolism, amino acid metabolism, lipid metabolism, and other amino acid metabolisms. These results indicate that LH023 could enhance the participation of intestinal microorganisms in metabolism and promote the growth of grass carp. Safety and non-pathogenicity are important indicators for the evaluation of probiotics [44]. Therefore, before the production and application of probiotics, the safety of the strain must be accurately evaluated. In this experiment, *A. hydrophila* challenge assay was performed, and the HE results showed that the intestinal tract, liver, head kidney, and mesonephric of grass carp in LH023 feeding groups did not exhibit any obvious lesions, and supplementing LH023 to feed significantly increased the immunoprotective ability against *A. hydrophila invasion*.

In summary, this study isolated *B. velezensis* from the intestines of grass carp and named it LH023. The results revealed that LH023 showed a strong antibacterial activity in vitro. The diets of LH023 enhanced the growth performance of grass carp, modulated the antioxidant system and inflammation response, improved intestinal health through regulating intestinal microbial community structure, and increased the grass carp's resistance to pathogen infection. This study demonstrates the potential application of LH023 in aquaculture, offering a promising strategy for the prevention and control of aquatic diseases.

## Figures and Tables

**Table 1:** The primers used in this study.

Primer	Sequence (5′-3′)
*16S rDNA-F*	AGAGTTTGATCCTGGTCAGAACGAACGCT
*16S rDNA-R*	TACGGCTACCTTGTTACGACTTCACCCC
*gyrB-bv-F*	TCTGCTTTCAAACCGTGTCC
*gyrB-bv-R*	TCAACCGTTATGCCGTCTTT
*IFN-γ2-F*	TGTTTGATGACTTTGGGATG
*IFN-γ2-R*	TCAGGACCCGCAGGAAGAC
*c-Rel-F*	GCGTCTATGCTTCCAGATTTACC
*c-Rel-R*	GCGTCTATGCTTCCAGATTTACC
*NF-кBp52-F*	TCAGTGTAACGACAACGGGAT
*NF-кBp52-R*	ATACTTCAGCCACACCTCTCTTAG
*IKKβ-F*	GTGGCGGTGGATTATTGG
*IKKβ-R*	GCACGGGTTGCCAGTTTG
*IKKγ-F*	AGAGGCTCGTCATAGTGG
*IKKγ-R*	CTGTGATTGGCTTGCTTT
*TLR-4-F*	GAATAATGGGCAGCCGTAAAGTC
*TLR-4-R*	TCCTCTCTTCCACATCTTCCAGA
*S6K1-F*	TGGAGGAGGTAATGGACG
*S6K1-R*	ACATAAAGCAGCCTGACG
*IL-4-F*	AATAGGGATCAACGAGAA
*IL-4-R*	TGAATGGTTATGTAGGGT
*IL-6-F*	ACTTTCGCAATCACAAT
*IL-6-R*	GATGTTTCAGATAACTCCC
*IL-8-F*	GAGTCTTAGAGGTCTGGGTG
*IL-8-R*	CAGGTTAAAATATTGTGCAT
*IL-10-F*	TTTGAGTTTGCCACCA
*IL-10-R*	ATGCCAGATACTGTTCG
*IL-15-F*	GCGAGAGGCTGAGGAAGTTT
*IL-15-R*	*GCGAGAGGCTGAGGAAGTTT*
*IL-17D-F*	GTGTCCAGGAGAGCACCAAG
*IL-17D-R*	GCGAGAGGCTGAGGAAGTTT
*TNF-α-F*	GCTGCTGTCTGCTTCACGC
*TNF-α-R*	AGCCTGGTCCTGGTTCACTCT
*Keap-1-F*	TTCCACGCCCTCCTCAA
*Keap-1-R*	TGTACCCTCCCGCTATG
*SOD-F*	TCCGCACTTCAACCCTTACAG
*SOD-R*	ACTTTCCTCATTGCCTCCCTT
*CAT-F*	GCCATCTCCAACGGCAACTT
*CAT-R*	CCAGACCTTAGTCAAATCAAACGG
*GSH-F*	AGGAGTTCCGAGATGTTGGATTC
*GSH-R*	GTCTCCATTCACATCCACCTTCT
*Claudin-12-F*	CTAAGGGGCACCTGCTACA
*Claudin-12-R*	TGGGGTGTTCACAGTTGTTT
*ZO-2-F*	TACAGCGGGACTCTAAAATGG
*ZO-2-R*	TCACACGGTCGTTCTCAAAG
*ZO-3-F*	TTGTCATTTTGGGTCCTCTG
*ZO-3-R*	CTATCCGCCTAACCGTGTC
*β-actin-F*	ACCCACACCGTGCCCATCTA
*β-actin-R*	CGGACAATTTCTCTTTCGGCTG

**Table 2:** The physiological and biochemical test results of LH023.

Number	Project	Results	Number	Project	Results
1	Sucrose	+	10	Hydrogen sulfide	−
2	Seven leaf glycosides	+	11	Raffinose	−
3	Mannitol	−	12	Simonds citrate	−
4	Inulin broth	+	13	Maltose sugar	−
5	Xylose	−	14	Sorbitol	−
6	Cellobiose	+	15	Arabinose	−
7	Indole experiment	−	16	Lactose	−
8	Methyl red	−	17	Gelatin experiment	+
9	V–P	−	—	—	—

**Table 3:** Results of drug susceptibility test of strain LH023.

Antibiotics	The judgment standard of inhibition zone diameter (mm)			Contents (μg)	Inhibition zone diameter (mm)
	Resistant	Intermediately sensitive	Sensitive		*Bacillus velezensis* LH023
Penicillin G	≤13	13–16	≥17	10	26.21^s^
Ampicillin	≤13	13–16	≥17	10	24.35^s^
Kanamycin	≤13	14–17	≥18	30	23.9^s^
Neomycin	≤12	13–16	≥17	30	24.95^s^
Erythromycin	≤13	14–22	≥23	15	26.89^s^
Midecamycin	≤13	14–17	≥18	30	30.32^s^
Norfloxacin	≤12	13–16	≥17	10	21.81^s^
Compound new ming	≤12	13–16	≥17	100	27.14^s^
Vancomycin	≤14	15–16	≥17	30	20.38^s^
Fluorine benzene ni	≤12	13–17	≥18	30	30.47^s^
Cephalexin	≤14	13–17	≥18	30	37.03^s^
Enrofloxacin	≤12	13–15	≥16	30	32.87^s^

*Note:* “S” stands for highly sensitive; “I” means moderately sensitive; “R” means insensitive.

## Data Availability

The data that support the findings of this study are available from the corresponding author upon reasonable request.
